# Multimodal Machine Learning Approaches to Detect Attention-Deficit/Hyperactivity Disorder (ADHD) Using Physiological and Behavioural Biomarkers: A Systematic Review

**DOI:** 10.3390/healthcare14193259

**Published:** 2026-10-01

**Authors:** Agmasie Damtew Walle, Ghazal Bargshady

**Affiliations:** 1 Department of Health Informatics, Debre Berhan University, Debre Birhan P.O. Box 445, Ethiopia; 2 Research Centre for Intelligent Computing and Systems, University of Canberra, Canberra, ACT 2617, Australia

**Keywords:** multimodal, machine learning, ADHD, systematic review

## Abstract

**Background**: Attention Deficit Hyperactivity Disorder (ADHD) is a common neurodevelopmental disorder characterised by persistent symptoms of inattention, impulsivity, and hyperactivity that can affect individuals throughout childhood and adulthood. Accurate and timely detection is important for appropriate support and management, but current assessment relies heavily on behavioural observations, clinical assessments, and information from multiple sources, which can be time-consuming and may be influenced by subjective judgement. These challenges have increased interest in machine learning (ML) and deep learning (DL) approaches that can analyse multiple physiological and behavioural data sources. Multimodal AI approaches may provide a more comprehensive and objective way to detect ADHD by combining complementary information from different data types. **Objectives**: This systematic review aimed to examine the use of ML and DL methods for ADHD detection using multimodal physiological and behavioural biomarkers, with particular attention to data sources, modelling approaches, multimodal fusion strategies, and reported diagnostic performance. **Methods**: A systematic review was conducted in accordance with the Preferred Reporting Items for Systematic Reviews and Meta-Analyses (PRISMA) 2020 guidelines to identify studies published from 2015 to 2026 that applied machine learning (ML) or deep learning (DL) methods to multimodal data for ADHD detection. We selected studies using predefined inclusion and exclusion criteria and extracted and synthesised data from eligible studies. The review included 25 studies. The multimodal data sources examined included neuroimaging modalities, such as functional magnetic resonance imaging (fMRI) and magnetic resonance imaging (MRI); physiological signals, including electroencephalography (EEG), heart rate variability (HRV), and electrodermal activity (EDA); behavioural assessments; and wearable or Internet of Things (IoT)-based measures. We also assessed the included studies for methodological quality and model performance to identify current evidence, limitations, and future research needs. **Results**: The included studies varied substantially in design and sample size, with participant numbers ranging from 22 to 15,883. Most studies used cross-sectional designs. Early multimodal fusion approaches frequently reported diagnostic accuracies above 80%, demonstrating the potential value of combining complementary physiological and behavioural information. Reported informative biomarkers included frontal brain activity, heart rate patterns, and oculomotor behaviour. However, substantial differences in data sources, acquisition procedures, feature extraction, modelling techniques, and evaluation protocols limited direct comparison across studies. Small sample sizes and a lack of standardised data collection and methodological protocols also challenged the generalisability and reliability of the reported findings. **Conclusions**: Multimodal machine learning and deep learning approaches show considerable potential for improving ADHD detection by integrating complementary physiological and behavioural information. However, substantial heterogeneity in data collection, devices, datasets, validation procedures, and reporting limits comparability and generalisability across studies. Future research should prioritise standardised data-collection protocols, larger and more diverse datasets (including children, adolescents, and participants from different cultural backgrounds), leakage-safe validation, and independent multi-site testing. Fusion strategies should be selected based on modality compatibility and study objectives. Improving model interpretability alongside predictive performance will also support clinical trust and real-world adoption.

## 1. Introduction

Attention Deficit Hyperactivity Disorder (ADHD) is a complicated mental condition that starts in childhood and continues into adulthood for many people, causing problems with focus, impulsive behaviour, and overactivity [1]. ADHD often goes undiagnosed because its symptoms are similar to other mental health problems, and diagnosis relies on personal judgment. Traditional tools such as doctor interviews and self-reporting may be prone to bias and inconsistency [2]. Advances in artificial intelligence and new approaches to integrating multiple data sources create an opportunity to improve ADHD diagnosis. By combining multiple biological signs with machine learning (ML) models, these methods aim to overcome the limits of using only one data type and improve diagnostic accuracy. However, it is still unclear how well these models perform in real medical settings and how scientifically reliable they are.

Several unimodal and multimodal approaches exist for automated ADHD detection. These methods utilise a variety of physiological and behavioural biomarkers. Key physiological biomarkers include photoplethysmography (PPG), electrocardiography (ECG), electroencephalography (EEG), and functional near-infrared spectroscopy (fNIRS). Behavioural biomarkers involve analysis of head and body pose or movement, as well as facial expressions. Integrating these diverse biomarkers enhances the accuracy and real-time detection capability of AI-driven ADHD diagnostic systems [2]. Unimodal methods, however, face critical reliability issues, including sensor failure and data quality inconsistencies [3]. For example, the measurements are prone to noise and inter-subject variability, and facial expression analysis may be unsteady under occlusions or with differing eye gaze and facial expressivity [4]. Such limitations highlight the need for better and more robust multimodal methods that can contribute significantly towards improving the diagnosis of ADHD.

Current ADHD prediction models have serious problems, including a lack of external validation, a high risk of bias, and low clinical utility. A systematic review found that only 7% were externally validated and 67% were classified as having a high risk of bias [5]. In addition, the dominance of diagnostic behaviouralistic models (88%) compared to treatment response and long-term outcome prediction models indicates a deficiency in models for incorporation into treatment planning and clinical decision-making. Generalizability is also an issue, as several studies report evidence of overfitting [2,5,6].These problems suggest a need for more sophisticated modelling techniques, better data, and research with larger sample sizes.

Multimodal approaches that combine various kinds of data can enable real-time, accurate diagnoses that better reflect the complexity of ADHD than unimodal approaches. However, constructing such models involves several challenges, including combining heterogeneous data, small sample sizes and dataset imbalances, non-standardised feature selection, and limited generalizability. Therefore, this systematic review summarises the scientific literature on constructing ML-based multimodal models for ADHD diagnosis and defines and discusses the issues involved. Physiological biomarkers and sensors hold immense promise for improving ADHD assessment. Approaches such as fMRI, EEG, eye tracking, HRV, and EDA provide tangible measures of the neurological and behavioural activity related to ADHD. These approaches help identify neurological, cognitive, and autonomic markers that conventional assessment processes cannot detect. Wearable and IoT technologies also enable the continuous assessment of these markers in real-world settings [7].

Despite these advances, traditional ways of identifying ADHD still face challenges because symptoms vary widely, sample sizes are small, and combining different types of data is limited. Artificial intelligence (AI), especially machine learning (ML) and deep learning (DL), can help by analysing complex, varied datasets to find patterns and signs of ADHD. AI-based models can improve diagnostic accuracy, reduce reliance on personal judgment, and potentially enable earlier detection [8]. Additionally, these models can combine data from behaviour, physiological functions, and environmental sensors to provide a more detailed and personalised ADHD diagnosis [9].

This systematic review was motivated by the traditional assessment approach to ADHD detection and by the limitations of artificial intelligence that uses unimodal data. Multimodal machine learning offers promise by combining behavioural, biological, and physiological biomarkers, which may provide a fuller understanding of ADHD traits; however, existing studies are scattered across different data types, datasets, modelling, and evaluation methods, making it hard to understand the current evidence and best methods. Consequently, this systematic review aims to identify and synthesise existing studies that use ML-based multimodal models to diagnose ADHD, examine the multimodal data sources used, assess the methodological quality of the models and fusion techniques, and highlight key challenges and research gaps to inform future model development and clinical integration. Multimodal approaches improve diagnostic accuracy and reliability and often surpass unimodal models in these respects [10]. Therefore, this systematic review aims to identify and analyse studies using multimodal ML models for diagnosing ADHD, including the unimodal data sources used and the quality of the models and their fusion.

Despite growing interest in using machine learning (ML) and deep learning (DL) methods with different data types for ADHD diagnosis, no complete summary exists of how well these methods perform across data types, and the challenges preventing their use in clinics remain unclear. This review looks at the following research questions:

- What ML/DL models have been applied to multimodal data for ADHD diagnosis, and how do they perform?
- What methodological limitations related to data heterogeneity, sample size, and standardisation affect the reliability of these models?
- What future research directions and methodological improvements could enhance the generalisability, robustness, interpretability, and potential clinical applicability of multimodal AI-based tools for ADHD detection?

## 2. Materials and Methods

This section presents the methodology used for a systematic literature review that aims to find, select, and critically assess studies relevant to ADHD diagnosis. We conducted a systematic review following PRISMA 2020 guidelines. We conducted an extensive literature search using databases such as PubMed, Scopus, and IEEE Xplore. The literature search covered study done in 2015 to 2026. Studies included in the systematic review presented original research using ML/DL techniques to identify ADHD in children and adults based on multimodal datasets. We excluded original studies without multimodal data and studies for which we did not have full-text access. We performed article selection, data extraction, and quality assessment independently using the modified Newcastle–Ottawa Scale. We extracted data in Microsoft Excel and managed references in EndNote v20. We narratively synthesised the results due to their heterogeneity.

### 2.1. Protocol and Registration

The protocol for this systematic review was prospectively registered with the International Prospective Register of Systematic Reviews (PROSPERO). PROSPERO assigned the registration number CRD420261509501. The PROSPERO registration provides details of the planned review methodology, including the review objectives, eligibility criteria, search strategy, study selection, data extraction, and planned analysis methods.

### 2.2. Study Design and Reporting

This systematic review examined recent artificial intelligence (AI)-driven multimodal approaches for ADHD detection and was conducted and reported in accordance with the Preferred Reporting Items for Systematic Reviews and Meta-Analyses (PRISMA) 2020 guidelines. We used the PRISMA 2020 checklist to support transparent and complete reporting of the review methods, study selection process, and findings. We followed a structured search and screening process to identify relevant studies, which we assessed against predefined inclusion and exclusion criteria. The PRISMA flow diagram summarises the study selection process, including identification, screening, eligibility assessment, and final inclusion. This approach provides a transparent framework for documenting how the evidence was identified and selected for inclusion in the review [11]. The review synthesised existing evidence on the application of machine learning (ML) and deep learning (DL) approaches to detect Attention Deficit Hyperactivity Disorder (ADHD), with a particular focus on studies using multimodal data sources. These approaches integrate information from different types of physiological and behavioural data, such as electroencephalography (EEG), heart rate, neuroimaging, cognitive tests, reaction time, and eye-tracking measures. By combining complementary information from multiple modalities, multimodal approaches aim to improve the accuracy and robustness of ADHD detection compared with single-source approaches.

### 2.3. Eligibility Criteria

This systematic review included studies that met predefined eligibility criteria. The study population comprised children and adults with ADHD, as well as participants identified as being at risk of developing ADHD where applicable. The primary focus was on studies that applied machine learning (ML) or deep learning (DL) algorithms to detect or classify ADHD using multimodal data sources. These data sources included combinations of physiological and behavioural measures, such as EEG, heart rate, neuroimaging, cognitive performance, reaction time, and eye-tracking data.

Eligible studies were original research, including experimental and observational studies, that investigated the application of ML or DL methods to multimodal ADHD detection or classification. Only articles published in English were included to maintain consistency in the assessment and synthesis of the available evidence. The literature search covered studies published from January 2015 onwards. We excluded studies that did not apply ML or DL methods, were not original research articles (systematic reviews, narrative reviews, editorials, letters, or conference abstracts), or focused exclusively on unimodal data. We also excluded studies that did not address ADHD detection or classification using multimodal physiological and/or behavioural information.

### 2.4. Search Strategy

We conducted a systematic search in the following three databases: IEEE, PubMed, and Scopus. We searched for articles published from January 2015 onwards. The search terms included combinations of MeSH terms and keywords using Boolean operators as follows:(“Attention Deficit” OR ADHD OR “Attention Deficit Hyperactivity Disorder” OR AD OR “Attention Disorder”) AND (“Machine Learning” OR “Deep Learning” OR ML OR DL OR LLM OR SVM OR “Support Vector Machine” OR “Random Forest” OR KNN OR “K-Nearest Neighbors” OR “Decision Tree” OR “Neural Network”) AND (multimodal OR multimodality OR “multi-modal” OR “multi-modality”) AND (physiological OR physiology OR behavioural OR behavioural OR “EEG” OR “fMRI” OR “Eye Tracking” OR “Wearable Devices” OR “Sensors”) AND (“Diagnosis” OR “Recognition” OR “Classification” OR “Detection” OR “Prediction”).

### 2.5. Study Screening and Data Extraction

Two independent reviewers screened the titles and abstracts of all identified studies and subsequently assessed the full texts of potentially eligible articles. Reviewers discussed and resolved disagreements through consensus. When consensus could not be reached, a third reviewer made the final decision. We extracted data using a standardised Microsoft Excel spreadsheet to promote consistency and accuracy throughout the data-extraction process. The extracted information included the authors and year of publication, characteristics of the study population, types of data and biomarkers used (e.g., EEG, fMRI, and behavioural measures), ML and DL models or algorithms applied, reported performance metrics (e.g., accuracy, precision, recall, sensitivity, specificity, and F1-score), and the main findings and implications of each study.

### 2.6. Quality Assessment

We assessed the methodological quality of all included studies using a modified Newcastle–Ottawa Scale (NOS) adapted to observational and AI-based modelling studies. The assessment covered several key domains, including sample representativeness, appropriateness of the ML or DL methods, clarity and completeness of model performance reporting, validity of outcome definitions, and use of appropriate model validation procedures, such as cross-validation. Each study received a total quality score ranging from 1 to 9. We classified studies scoring 7–9 as high quality, 4–6 as moderate quality, and 1–3 as low quality. These categories provided a consistent framework for evaluating the methodological quality and reliability of the included studies and supported interpretation of the review findings [12]. Although we excluded no study solely for quality, the quality score reflected the rigour of the included studies. Thus, we explicitly determined the methodological quality of the studies. Supplementary Table S1 presents the quality assessment results for the included studies.

### 2.7. Data Synthesis and Analysis

We systematically reviewed the computational approaches used across studies, spanning traditional machine learning techniques such as Support Vector Machines (SVMs), Random Forest (RF), k-Nearest Neighbors (k-NN), and Logistic Regression, as well as more recent deep learning architectures, including Convolutional Neural Networks (CNNs), Recurrent Neural Networks (RNNs), and hybrid or ensemble models. This allowed us to track trends in algorithm selection and identify which approaches were most frequently applied to ADHD classification tasks. Studies varied considerably in the neurophysiological, neuroimaging, and behavioural data types used to train and validate their models. These included electroencephalogram (EEG) signals, functional magnetic resonance imaging (fMRI) data (both resting-state and task-based), structural MRI, behavioural and cognitive assessment scores, and, in some cases, combinations of multiple data modalities (multimodal fusion approaches) [13]. Organising these data types enabled us to compare how different input sources influenced model design and diagnostic performance.

For each study, we extracted reported performance metrics, including accuracy, precision, recall (sensitivity), specificity, and F1-score, along with area under the curve (AUC) values where available. This allowed a qualitative comparison of how well different modelling approaches performed across varying datasets and diagnostic contexts, while acknowledging that differences in validation procedures, sample characteristics, and metric reporting standards limited direct statistical comparison. We also examined each study’s methodological features, including sample size, participant age range, diagnostic criteria used to define ADHD cases, comparison/control groups, cross-validation strategies, and whether the study performed external validation. This contextual information was essential for interpreting reported performance metrics appropriately and for assessing the robustness and generalisability of each study’s findings.

By synthesising information across these four domains, this systematic review approach enabled a meaningful comparison of modelling techniques and their diagnostic performance for ADHD, despite considerable variability in study design, data types, and reportings practices. This method yielded valuable insights into the current state of ML/DL applications in ADHD diagnosis, while also highlighting gaps and inconsistencies that meta-analytic pooling would have obscured.

### 2.8. Ethical Consideration

As this systematic review relies solely on previously published research, it did not involve direct human or animal participation, and therefore did not require ethical approval. The review followed standard systematic review reporting guidelines (PRISMA) to ensure methodological transparency.

## 3. Results

This section presents the principal findings of the systematic review. It begins with a summary of the study selection process, outlining how relevant studies were identified, screened, and included, typically illustrated through a PRISMA flow diagram detailing the number of records identified, screened, excluded, and ultimately retained for full review. This is followed by a descriptive overview of the included studies, summarising key characteristics such as sample sizes, participant demographics, the types of input data utilised, and the range of machine learning and deep learning methods applied across the literature. Building on this foundation, the section then narrows its focus to a detailed examination of multimodal artificial intelligence approaches that integrate two or more data modalities and critically evaluates their effectiveness in detecting and classifying ADHD, highlighting patterns in diagnostic performance, methodological strengths, and limitations associated with these integrative approaches.

### 3.1. Search Result

The systematic search of relevant databases yielded an initial pool of 514 papers. Before formal screening, we pre-screened records to remove clearly ineligible items and excluded 225 papers. This included 126 duplicate records identified across databases, 61 papers flagged as ineligible through automated screening tools, and 38 papers excluded for other pre-specified reasons (non-English language publications, incorrect document type, or irrelevance matter). After this initial filtering stage, 289 papers remained and proceeded to title and abstract screening, during which 154 papers were excluded for failing to meet the review’s basic inclusion criteria.

**Figure 1:**
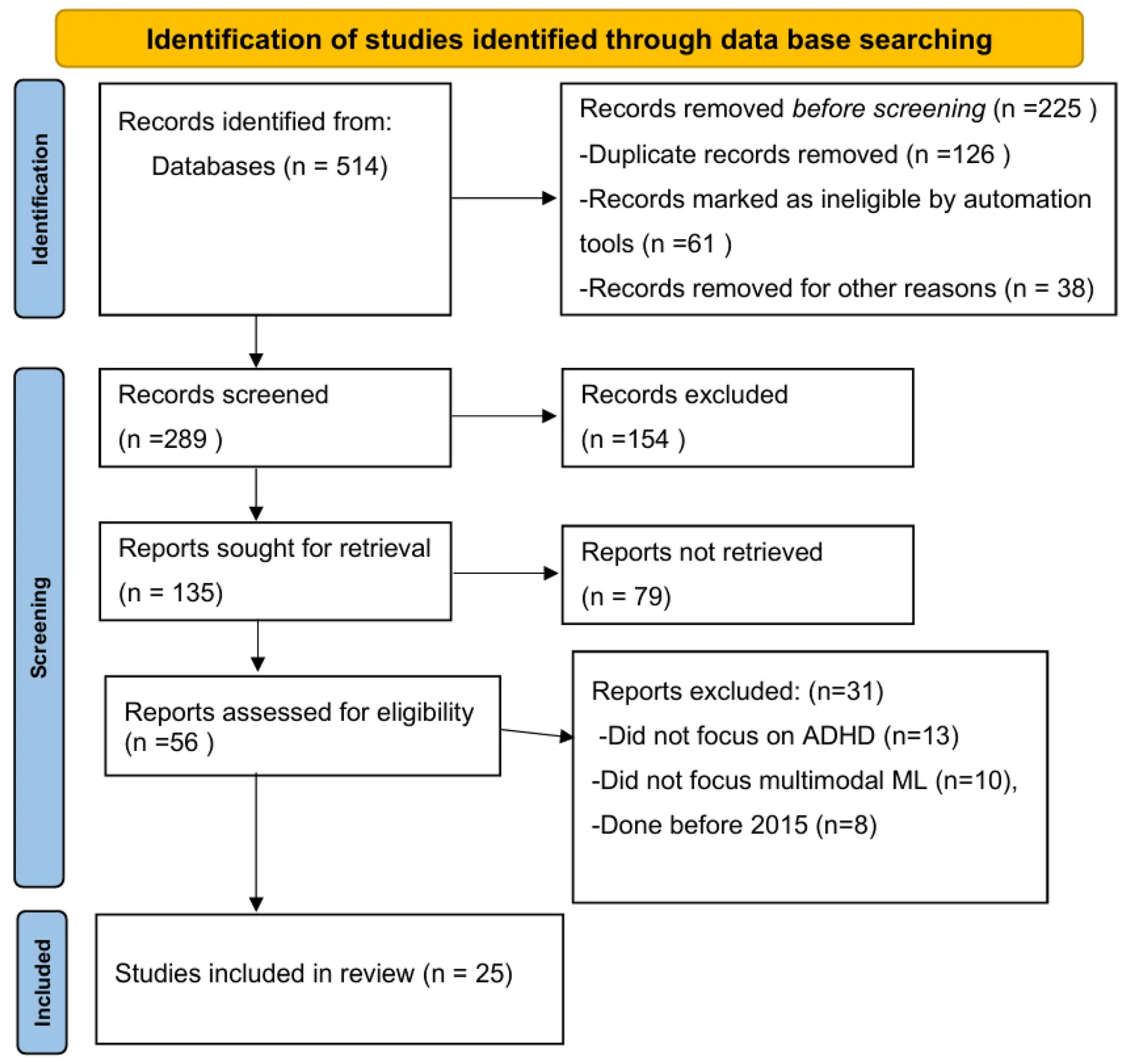
PRISMA flow chart showing the study selection process of articles for a systematic review of computational AI to detect ADHD.

This left 135 papers deemed potentially eligible, for which we attempted full-text retrieval. However, we could not obtain the full texts of 79 papers because of restricted access, unavailability through institutional subscriptions, or failure to respond to author contact requests. Consequently, we successfully retrieved 56 papers and subjected them to detailed full-text eligibility assessment. Of these, 31 papers were excluded for the following reasons: 13 papers did not specifically investigate Attention Deficit Hyperactivity Disorder (ADHD), 10 papers did not employ multimodal machine learning approaches (they relied on a single data modality rather than integrating multiple data types), and 8 papers were published prior to 2015 and therefore fell outside the review’s specified timeframe. After this final round of exclusions, 25 studies met all eligibility criteria and were included in the systematic review for qualitative synthesis and analysis (Figure 1).

### 3.2. Characteristics of Included Studies

Table 1 summarises the key characteristics of the 25 studies included in this review, including authors, year of publication, country, data source, population, sample size, and modalities used for ADHD detection. These studies varied considerably in design and scope, employing a wide range of data types, including neuroimaging, EEG, behavioural, physiological, and multimodal data, across diverse populations spanning children, adolescents, and adults.

Across the 25 included studies, participant age composition varied, with three distinct patterns observed. Most studies, 14 of 25 (56%), focused exclusively on adult populations, using samples drawn from adult participants diagnosed with or screened for ADHD. A smaller subset, 6 of 25 studies (24%), focused exclusively on paediatric populations, examining children diagnosed with or assessed for ADHD. The remaining 5 studies (20%) adopted a mixed-age design, including both child and adult participants within the same study sample.

Regarding the geographical distribution of the included studies, the 25 studies reviewed were conducted across 11 countries, spanning North America, Europe, and Asia. The United States contributed the largest proportion of publications, with 8 studies (32%), followed by the United Kingdom with 4 studies (16%). China, India, Germany, and Korea each contributed two studies, representing 8% of the total sample. The remaining five countries—Iran, Austria, Brazil, Italy, and France—each contributed one study, representing 4% of the total sample. Together, these 11 countries account for all 25 included studies (100%), with the United States and the United Kingdom alone contributing 12 studies (48%) of the reviewed literature.

**Figure 2:**
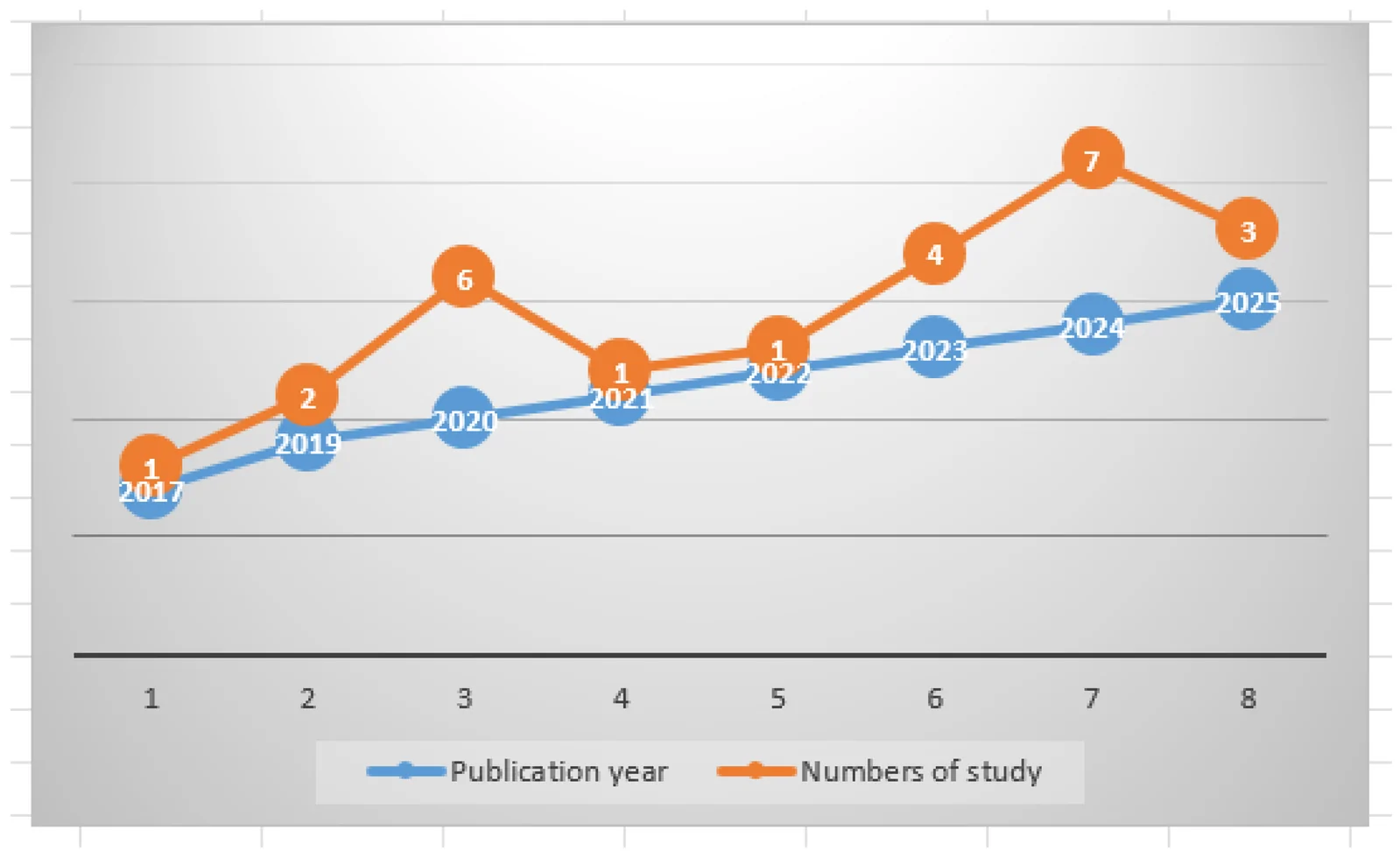
Publication year distribution.

Research in this domain started slowly, with only one study in 2017, and remained low through 2021 and 2022. Interest in the topic began to rise from 2020 onward, peaking in 2024, when seven studies were published. The trend upward suggests growing attention and continued participation in this research area (Figure 2).

Regarding study design, most included studies used a cross-sectional design, assessing ADHD-related traits and biological markers such as physiological measures and neuroimaging data at a single time point. These cross-sectionalwith healthy control participants, aiming to identify distinguishing differences across various data modalities, including EEG, fMRI, and behaviouralarious data modalities, including EEG, fMRI, and behavioural measures. A smaller subset of studies adopted a longitudinal design, following participants over time to examine how these measures changed and how they related to the progression or persistence of ADHD symptoms; however, such designs were comparatively less common across the reviewed literature. Sample sizes across the included studies varied substantially, ranging from as few as 22 participants to as many as 15,883 participants, reflecting considerable heterogeneity in study scale and statistical power. The primary population of interest across the reviewed studies was individuals diagnosed with ADHD, with a majority of studies additionally incorporating healthy control groups to enable direct comparison between clinical and non-clinical populations.

Across the included studies, structural MRI and functional MRI were the most frequently used data types, each appearing in six studies, followed closely by behavioural or task performance measures, also used in six studies. Diffusion MRI (DTI) and EEG were used in five studies each. In contrast, several other data types including electroretinogram (ERG), magnetoencephalography (MEG), positron emission tomography (PET), environmental exposure data, and parental reports appeared far less frequently, in only a few studies. This distribution indicates that while neuroimaging modalities (particularly structural and functional MRI) and behavioural assessments dominate the current body of ADHD detection research, alternative data sources such as ERG, MEG, PET, and environmental or parental report data remain comparatively underexplored, suggesting potential avenues for future investigation [1,2,7,13,18].

**Figure 3:**
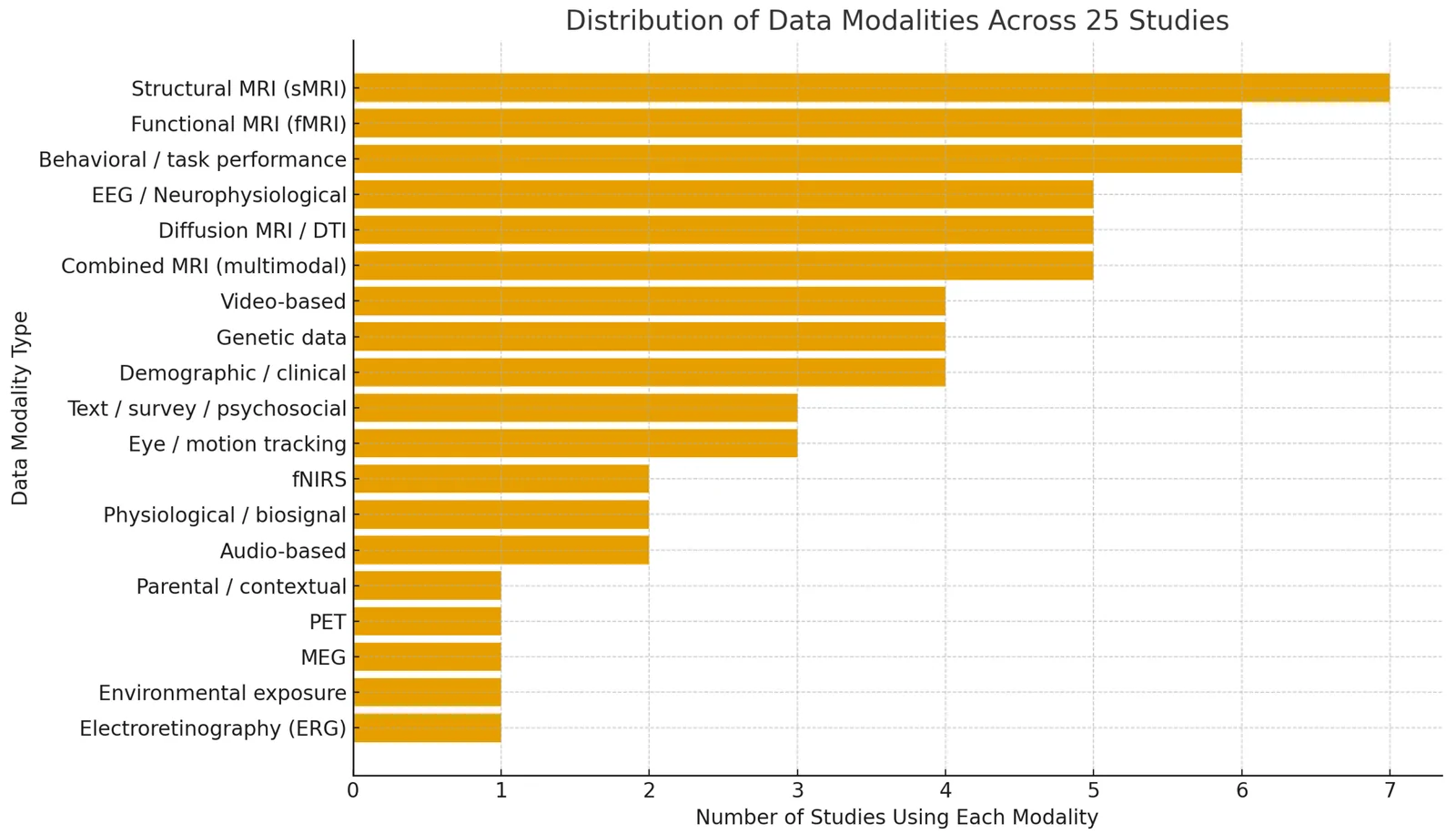
Data modality distribution.

For data collection, several studies used clinical interviews and standardised diagnostic tools, such as the ADHD Rating Scale, to confirm ADHD diagnoses and ensure diagnostic accuracy. In addition, several studies used real-world, ecologically valid data collection approaches, using sensors and wearable devices to monitor bodily activity and physiological responses during participants’ daily activities and capture naturalistic insights into ADHD-related symptoms and behaviours outside controlled settings. Conversely, other studies relied on controlled laboratory environments, employing brain imaging techniques and structured behavioural observation protocols to collect data under standardised experimental conditions. Table 2 summarises machine learning models and architectures, evaluation metrics, multimodal fusion methods, and key outcomes reported in the 25 included included studies (Figure 3).

**Figure 4:**
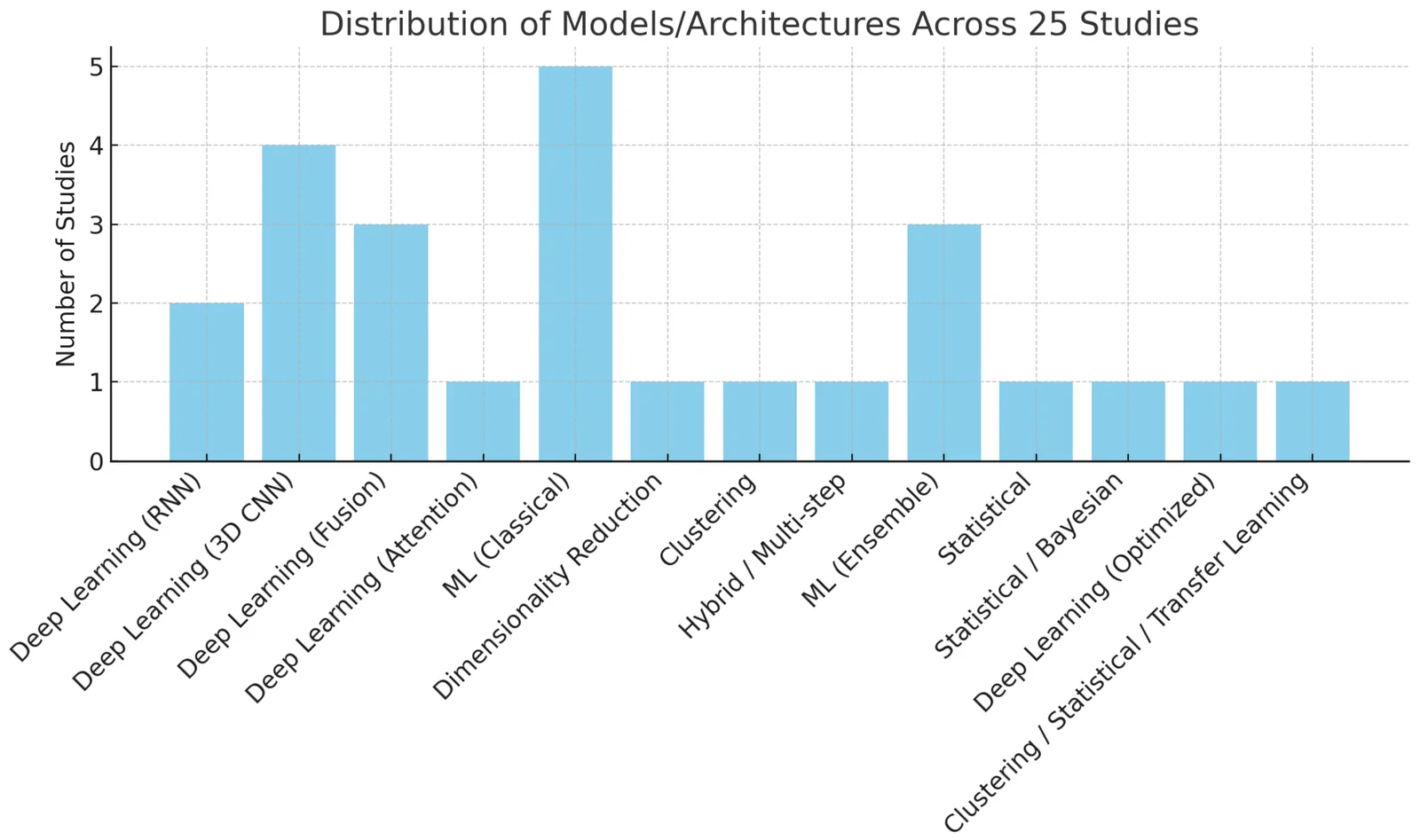
Model/architecture distribution.

Regarding computational approaches, the included studies applied a diverse range of machine learning models. Traditional machine learning algorithms commonly employed included Support Vector Machines (SVMs), Random Forest (RF), k-Nearest Neighbors (k-NN), and Logistic Regression (LR). In addition, several studies utilised more advanced deep learning architectures, including Convolutional Neural Networks (CNNs) and Long Short-Term Memory networks (LSTMs), reflecting a growing trend toward leveraging sophisticated modelling techniques capable of capturing complex, high-dimensional patterns within neuroimaging, physiological, and behavioural data for ADHD detection [2,4,6,7,9,16] (Figure 4).

**Figure 5:**
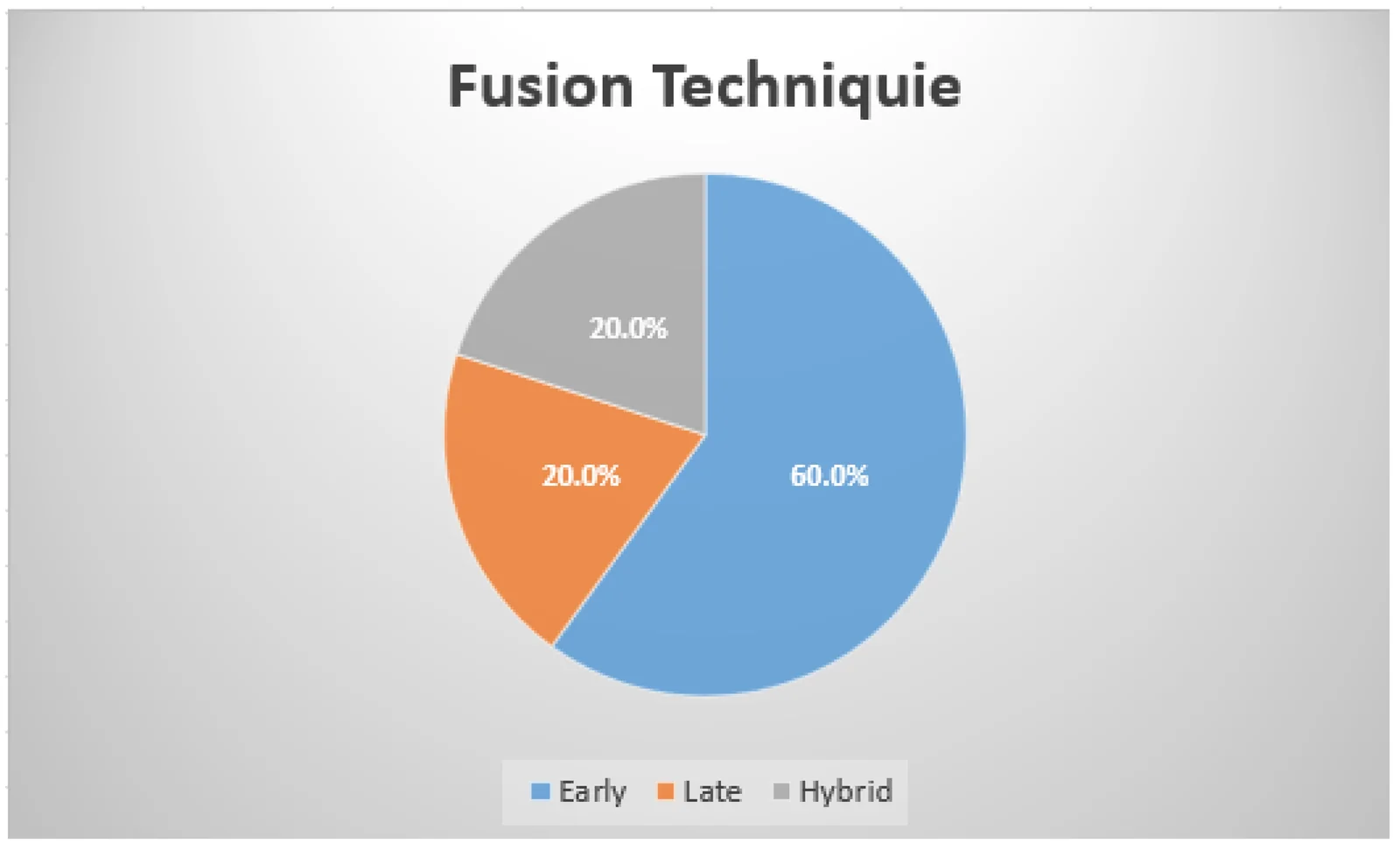
Model/architecture distribution.

Among the studies, many of the included studies explored multimodal data fusion techniques as a means of enhancing model performance and diagnostic accuracy, reflecting a growing recognition that integrating multiple data sources can capture complementary aspects of ADHD related neural, physiological, and behavioural patterns. Among the various fusion strategies employed, early fusion, which involves integrating features from multiple modalities at the input stage, prior to classification, emerged as the most commonly used approach across the reviewed literature [1,2,13,16,23,27,33] (Figure 5).

The included studies demonstrated that multimodal machine learning and deep learning approaches have considerable potential for ADHD detection and assessment. Across the studies, models integrating neuroimaging, physiological, behavioural, and other complementary modalities generally achieved moderate to high predictive performance, with several studies reporting diagnostic accuracies above 80%. Commonly reported effective approaches included Support Vector Machines, Random Forests, Convolutionastudies, models integrating neuroimaging, physiological, behavioural, and other complementary modalities generally achieved moderate to high predictive performance, with several activity, cortical connectivity, heart rate and physiological signals, oculomotor behaviour, and behavioural measures. Both early and late fusion strategies showed promising results, although performance varied by data modalities and modelling approaches. Multimodal models frequently outperformed unimodal approaches by integrating complementary information, but substantial variation in datasets, sample sizes, features, validation procedures, and evaluation metrics limits direct comparison across studies and highlights the need for larger datasets and standardised evaluation methods.

## 4. Discussion

This review combines current evidence on using machine learning and deep learning methods to diagnose ADHD across different data types. The discussion compares the main findings with existing studies, highlights the strengths and weaknesses of using multiple data types, and examines how AI-based methods could affect clinical practice and ADHD assessment research. It focuses on the benefits of combining data sources, finding important physical and behavioural signs, and the ongoing challenges that slow progress in this area.

### 4.1. Data Modalities in ADHD Detection

Each study used different data or data sources relevant to its task. In ADHD detection, researchers have explored multiple data modalities, such as physiological, imaging, and behavidentifying key physical and behavioural signs, and themation about the inherent symptoms of ADHD, and fusion can improve prediction accuracy.

#### 4.1.1. Physiological Data

Physiological information most often includes metrics of physiological states such as skin temperature, heart rate variability (HRV), and electrodermal activity (EDA). For example, one study used EDA, HRV, and skin while participants completed Stroop tasks to compare ADHD patients with healthy participants [2]. Physiological measurements from wearable devices can track precise bodily states during tasks, with considerable detail in monitoring attention and arousal. In most studies, researchers integrated physiological data with behavioural data to improve prediction [7].

#### 4.1.2. Imaging Data

Brain imaging techniques like fusuch as functional magnetic resonance imaging (fMRI), magnetic resonance spectroscopy (MRS), and electroencephalography (EEG) have been important in identifyingbased causes of ADHD. For example, one study used task-based fMRI along with behavioural results to show how combining brain scans with thinking tasks can help diagnose ADHD [23]. Similarly, diffusion tensor imaging (DTI) and structural MRI have been used to identify key features, such as how well certain brain areas, such as the right inferior frontal gyrus, function, to distinguish between ADHD patients and controls [26].

These neuroimaging modalities provide data on the brain’s anatomical and functional characteristics. Moreover, one study used functional near-infrared spectroscopy (fNIRS), a non-invasive method, combined with clinical assessment to predict ADHD in a group of medication-naïve ADHD patients [29]. Imaging data is typically costly and difficult to acquire in large samples, but it can detect potential ADHD biomarkers.

#### 4.1.3. Behavioural Data

Behavioural measures such as task performance (Go/No-Go tasks, Iowa Gambling Task), eye-tracking, and movement data have been extensively used to identify ADHD. As seen in [13], eye-tracking and actigraphy (activity and movement) were used to quantify attentional behaviours and hyperactivity. These measures are valuable for ADHD behavioural phenotype and are easily accessible in naturalistic settings. Behavioural data, while informative, often becomes even more valuable when combined with other modalities, as in a study that used raw RGB video to detect ADHD with 95.5% accuracy [7].

### 4.2. Model or Architecture

This systematic review found that researchers have used a wide range of machine learning (ML) and deep learning (DL) models to detect and classify ADHD from multimodal data. Models and architectures varied across studies depending on data type, volume, and analytical goals. Traditional machine learning methods, particularly Support Vector Machines (SVMs), were used primarily in studies that applied structured data such as MRI, EEG, and actigraphy. For example, refs. [1,26] applied SVMs to demonstrate satisfactory performance in ADHD classification from neuroimaging features. Such models are especially suited to loand their interpretable output can be valuable in clinical uset can be valuable in clinical use.

Over the past few years, more studies have focused on deep learning models, especially for handling complex and diverse data such as videos, audio recordings, and multiple sensor inputs. Models like 3D Convolutional Neural Networks (3D-CNNs), Long Short-Term Memory (LSTM) networks, and attention-based models have become popular [7]. 3D-CNNs learn spatial information from video data, and the AVEN (Attention-based Video Ensemble Network) model learns temporal and spatial patterns with a high classification rate (98.67%).

Several studies also employed ensemble learning and combined models with multiple algorithms to improve classification performance. These strategies guaranteed robust results while also making it easier to capture different features across modalities. A study by [20] used an ensemble model of Random Forest classifiers and Logistic Regression, and ref. [29] used a fusion model of audio–visual features flowing through attention-based networks. Despite these results, several problems remain. Deep learning methods usually need large amounts of labelled data, which was limited in some studies. Also, the complexity and hidden workings of DL models make them hard to understand, which is important for clinical decisions. Model performance depends on the type and quality of input data, so standard methods for collecting and validating data are needed.

### 4.3. Data Fusion Techniques

In ADHD detection, combining data from different sources has become a new way to improve diagnosis by bringing together information from brain scans, body signals, and behaviour [34]. Two main methods are often mentioned. Early fusion combines raw or processed data from different sources into one dataset before training, allowing models to learn combined features but potentially creating very large, noisy data. Late fusion combines results or predictions from each source-specific model, offering more flexibility, better handling of missing data, and broader applicability. Recent studies show that early fusion usually works better than using a single data type and helps identify reliable markers for ADHD diagnosis [1].

#### 4.3.1. Early Fusion

Early fusion combines features from multiple modalities into a single feature representation before the application of the machine learning or deep learning model. This strategy allows the model to learn relationships between modalities directly from the combined feature space. For example, ref. [1] applied early fusion to physiological data (BVP and GSR), motion data (acceleration), environmental data, and educational data to predict behavioural changes in students. Similarly, ref. [2] combined electrodermal activity, heart rate variability, and skin temperature features and applied machine learning models, including Logistic Regression and SVM, for ADHD classification, achieving 81.6% accuracy.

The main advantage of early fusion is its ability to capture interactions between different modalities within a unified feature space. It can also provide a relatively straightforward fusion framework when the modalities are compatible and can be appropriately aligned and scaled. However, combining heterogeneous features before model training can substantially increase the dimensionality of the input space. Early fusion may therefore be more sensitive to differences in scale, noise, missing data, and feature quality across modalities, potentially increasing the risk of overfitting, particularly when the available dataset is small. Thus, early fusion is most appropriate when the modalities can be reliably aligned and their combined feature representation is sufficiently informative for the prediction task.

#### 4.3.2. Late Fusion

Late fusion combines the outputs, predictions, or learned representations of models trained separately on individual modalities. Unlike early fusion, each modality can be processed independently using a model or feature-extraction strategy appropriate to its characteristics. This provides greater flexibility when modalities differ substantially in their data structure, dimensionality, or noise characteristics.

A study, ref. [7] employed a late-fusion strategy to combine models based on RGB video, facial landmarks, and body pose landmarks. Using Bi-Stream Fusion (BSF) and the ADHD Vision Ensemble Net (AVEN), the study reported 98.67% accuracy. This approach illustrates the potential of late fusion to integrate complementary information while allowing each modality to be modelled independently.

The principal advantage of late fusion is its flexibility. Modality-specific models can be optimised independently, which may help when modalities have substantially different characteristics. It can also reduce a noisy modality’s influence on another modality’s feature representation. However, late fusion may require additional models and therefore increase computational complexity. Its performance also depends on how effectively the individual models’ outputs are combined. Furthermore, because modalities are integrated later, some low-level interactions between modalities may not be captured as effectively as with early fusion.

#### 4.3.3. Comparison of Early and Late Fusion

This systematic review indicates that neither early nor late fusion is universally superior for ADHD detection. Their relative advantages depend on the modalities, the dataset, and the modelling task. Early fusion provides a direct mechanism for learning cross-modal relationships within a shared feature space and may be advantageous when modalities are well aligned and complementary. In contrast, late fusion provides greater flexibility by allowing modality-specific processing and may be more suitable when the modalities are heterogeneous or have substantially different noise and feature characteristics.

The reviewed studies also demonstrate that reported performance cannot be attributed to the fusion strategy alone. Differences in datasets, feature representations, machine learning algorithms, evaluation protocols, and sample sizes may also contribute to variations in classification performance. Therefore, the higher accuracy reported by some late- or early-fusion studies should not be interpreted as evidence that one fusion strategy consistently outperforms the other. Instead, fusion strategy should be selected according to the characteristics and compatibility of the available modalities, as well as the requirements of the ADHD detection task [2,7,29,35].

### 4.4. Sample Size, Overfitting, and Data Leakage

The included studies exhibited marked heterogeneity in sample size, ranging from as few as 6–22 participants to large-scale cohorts exceeding 5000 individuals. Most of the studies included fewer than 100 participants and several did not report sample size. This is a critical methodological concern: machine learning algorithms generally require sample sizes larger than the dimensionality of the feature set to avoid overfitting [36], and small, underpowered samples often yield inflated, unstable effect sizes and accuracy estimates due to sampling variability. This pattern has been empirically demonstrated in psychiatric neuroimaging, where studies with small samples have reported classification accuracies as high as 95%, while large-scale studies on the same diagnostic question yield accuracies closer to 60–65% [37,38]. Similar sample-size-dependent inflation of accuracy has been documented in schizophrenia neuroimaging classification and in multi-site depression classification [38,39]. This risk is compounded by inconsistent methodological safeguards across the reviewed studies. Feature selection was frequently performed on the full dataset prior to cross-validation splitting rather than nested within each training fold, a practice of double-dipping that introduces data leakage and systematically biases performance estimates upward [38,39]. Similarly, few studies clarified whether train/test splits were performed at the subject level, raising the risk that data from the same participant appeared in both partitions, further inflating reported accuracy [40] External validation on independent cohorts was also rarely reported, despite evidence that models performing well within a single dataset often generalize poorly to external or multi-site samples [39]. Collectively, these findings indicate that reported diagnostic accuracies in this field particularly from studies with fewer than 100 participants and without nested cross-validation or external validation—should be interpreted with caution and future work should prioritize larger multi-site datasets, leakage-safe nested validation pipelines, and independent external testing to improve reliability and generalizability.

### 4.5. Model Performance

In these studies, researchers used different scores to measure multimodal model performance, such as accuracy, precision, recall, F1 score, sensitivity, specificity, and area under the receiver operating characteristic curve (AUC). These scores help compare how well the model can tell apart ADHD patients from controls and correctly identify people with ADHD.

Studies used accuracy, precision, recall, and AUC to measure how well their ADHD Vision Ensemble Net worked, reaching 98.67% accuracy and 98.88% recall [7]. Similarly, a high accuracy of 95.5% was reached for identifying ADHD from raw RGB video [16]. A study by [29] got an AUC of 0.892, sensitivity of 80.28%, and specificity of 84.51% by combining fNIRS and clinical assessments early on. These scores provide a full picture of model performance, especially for clinical diagnoses where precision and recall must be high to reduce mistakes. AUC is often used as a secondary score to assess how well the model separates groups, especially when data is imbalanced.

A review of existing research on diagnosing ADHD showed that machine learning and deep learning models were used to get automated, accurate diagnoses, but their results were not final. This means model performance changes depending on the dataset. Also, the choice of feature selection method greatly affects the model’s results. Most experts say you need to adjust many settings to get the best results [5]. Changes to the data are expected to strongly affect how well these models work, showing they do not work equally well everywhere. Although these models help professionals make decisions, higher accuracy would make medical experts trust them more [41].

## 5. Conclusions

Although ADHD is not curable, if diagnosed properly and followed by care and treatment, the quality of life for ADHD patients can be significantly improved. This review offers a detailed evaluation of a multimodal data source. The paper primarily focuses on machine learning and deep learning methods that utilise multimodal data for computer-aided diagnosis. It covers the main models, feature engineering pipelines, experimental datasets, and the strengths and weaknesses of each approach. To navigate the current wealth of evidence, the survey outlines problems and limitations in current diagnostic methods and offers suggestions to address these challenges and improve outcomes. The early fusion technique was also an appropriate approach for detecting ADHD.

This paper aims to guide researchers using multimodal AI to detect ADHD by recommending suitable models and accessible datasets to help build more accurate, innovative diagnostic tools. The study followed a clear method for selecting articles, using databases and set inclusion and exclusion rules. To reduce bias and improve quality, several authors participated in the selection process. However, the authors may still have excluded important high-quality articles. Classification errors can also occur, especially when studies use more than one technology and may fit into multiple categories.

Based on the study and the revealed gaps, the authors indicate several directions to advance ADHD diagnosis beyond multimodal sources of information. First, standardising resting-state and task-based data is long overdue, especially when collected using multimodal data-source techniques, as current research largely targets task-based modalities while resting-state data remain comparatively under-researched. Standardisation would improve comparability and replicability across studies. In addition, heterogeneity in data-collection devices is a major challenge, and improving model generalizability and the consistency of diagnostic procedures is critical. Standard data-collection procedures can address this issue and allow stronger conclusions. Another critical point is the use of large-scale datasets.

Moreover, future studies should also address validation and reporting weaknesses identified in this review. Small and often unreported sample sizes, feature selection outside proper cross-validation, and limited external and limited external validation increase the risk of overfitting and inflated performance estimates; future work should therefore use leakage-safe nested cross-validation, clearly report subject- versus scan-level data splitting, and validate multi-site samples before claiming clinical utility. As this review found no consistent superiority of either early or late fusion, future studies should select fusion strategies based on modality compatibility rather than default preference. Given that most include Because most included studies focused on adult populations from a limited number of countries, more diverse, paediatric, and cross-cultural improve generalisability. Finally, future research should balance predictive accuracy with interpretability, as transparent models are more likely to gain clinical trust and support real-world adoption.

## Figures and Tables

**Table 1:** Characteristics of included studies (N = 25).

Authors, year	Country	Data Source	Population	Sample Size	Modalities Used
Nash et al. 2024 [7]	UK	The Intelligent Sensing ADHD Trial (ISAT)	Adults (18–53 years) with and without ADHD	22 (10 ADHD, 12 Control)	RGB video, facial landmarks, body pose, hand landmarks
Zhang et al. 2024 [14]	China	EEG-based neurophysiological data from tasks	Children with ADHD and typically developing children	81 (49 ADHD, 32 Control)	EEG, behavioural task performance
Abdolmaleki et al. 2020 [15]	Iran	ADHD-200 Dataset	Children, Adolescents	N/A	Structural and Functional MRI
Shuanglin et al. 2024 [16]	UK	Intelligent Sensing Laboratory dataset	Adults with ADHD	Not specified	Audio (speech), text, RGB video
Yichun et al. 2024 [10]	UK	Multimodal ADHD dataset, AVEC	ADHD and Depression patients	N/A	Audio, Video (facial), EEG, fMRI
Lylath et al. 2022 [17]	India	Various online resources	Children with ASD	N/A	Text, EEG, GSR, Robotics
S. Fazeli et al. 2023 [8]	USA	Smartwatch (Garmin vivoactive 4S)	College students (ex/active-duty military)	14	Physiological (HR, respiration, stress, activity)
W. Lin et al. 2023 [18]	UK	Audio–visual distress dataset	Adults (18–53 years)	106	Audio, Video (body/gaze/facial AUs), MFCCs
Kennedy et al. 2024 [19]	USA	ENIGMA, PGC, LIMBIC-CENC	Participants aged 15–90 years	15,883	Text (verbal learning/memory), demographics, diagnosis
Kautzky et al. 2020 [20]	Austria	PET imaging and SNP genotyping	Adults with ADHD and controls	38 (16 ADHD, 22 HC)	PET ([11C]DASB), Genetic (HTR1A/B, TPH2)
Andrikopoulos et al. 2024 [2]	USA	Wearable sensor data (Stroop tests)	Adults (ADHD and controls)	76 (32 ADHD, 44 HC)	EDA, HRV, Skin temperature
Menon et al. 2020 [21]	USA	Adolescent Brain Cognitive Development	Children aged 9–10 years	1100 (550 DBD, 550 TD)	MRI imaging, behavioural assessments, demographic features
De Lacy et al. 2023 [22]	USA	ABCD Study cohort	Children (9–12 years)	5777	Text (behavioural), MRI, parental and school data
McNorgan et al. 2020 [23]	USA	MTA follow-up MRI and behavioural data	Young adults	80	fMRI (Go/No-Go, IGT), behavioural
Muthuraman et al. 2019 [24]	Germany	EEG/MEG data	Adolescents	22 (11 ADHD, 11 HC)	EEG (64-ch), MEG (275 sensors)
Yoo et al. 2019 [25]	Korea	Multimodal MRI + genetic data	Child and adolescents	269 (191 ADHD, 78 TDC)	MRI, genetics
Luo et al. 2020 [26]	USA	Neuroimaging data	Young adults	72 (36 ADHD, 36 HC)	Structural MRI, DTI, fMRI
Manjur et al. 2025 [27]	USA	Electroretinogram (ERG) data	286 subjects (146 control, 94 ASD, 46 ADHD)	286	ERG (retinal response to light)
Yoo et al. 2020 [28]	Korea	Multimodal (clinical, genetic, neuroimaging)	ADHD youth	119 (83 train, 36 val)	Clinical, neuropsych, genetic, environmental, neuroimaging
Weibe et al. 2024 [13]	Germany	Multimodal: Eye tracking, EEG, actigraphy, behaviour	Adults with ADHD and controls	86 (50 train, 36 test)	Eye tracking, EEG, actigraphy, behavioural
Ma G et al. 2025 [29]	China	Clinical + fNIRS + oculomotricity	Young adults (18–28)	142 (71 ADHD, 71 HC)	Clinical interviews, fNIRS, CPT, oculomotricity
Chaim-Avancini et al. 2017 [30]	Brazil	MRI + DTI	Adults	133 (67 ADHD, 66 HC)	Structural MRI, DTI
Grazioli et al. 2021 [31]	Italy	Functional near-infrared spectroscopy	Children with ADHD	33 ADHD children	Clustering of treatment responses
Guigou et al. 2025 [32]	France	Eye-tracking, touch data, and emotion-tracking	Children	24 children aged 7–12 years	Multimodal biometry
Firdous et al. 2023 [33]	India	Structural MRI, DTI	Adults (stimulant-naïve, childhood-onset ADHD)	133 (67 ADHD, 66 HC)	MRI (T1), DTI

**Table 2:** Summaryof machine learning models and architectures used in the included studies.

Authors	Model/Architecture	Evaluation Metrics	Fusion Method	Key Results/Outcome
Nash et al. 2024 [7]	Landmark LSTM, ShuffleNet 3D CNN, Bi-Stream Fusion (BSF-WC, BSF-V), AVEN (ADHD Vision Ensemble Net)	Accuracy, Precision, Recall, F1 Score, AUC, Confusion Matrix	Late	AVEN achieved 98.67% accuracy, 98.01% precision, and 98.88% recall; BSF-WC and BSF-V also performed well; the Landmark model was more robust than ShuffleNet 3D.
Zhang et al. 2024 [14]	Deep Neural Network (DNN) fusion model	Classification accuracy (single-task vs. multi-task)	Hybrid	Multi-task EEG fusion achieved 89% detection accuracy vs. 81% for single-task; tasks with varying difficulty revealed attention-related deficits more clearly.
Abdolmaleki et al. 2020 [15]	Multi-modal 3D CNN	Accuracy, Classification score	Hybrid	LDA achieved 74.93% accuracy; early and late fusion improved accuracy by 5.84%.
Shuanglin et al. 2024 [16]	Action Recognition Network	Accuracy	Early	High accuracy of 95.5% for ADHD detection using raw RGB videos.
Yichun et al. 2024) [10]	Spatial–Temporal Attention Networks	Accuracy, Performance Comparison	Loss-level	Predicted over 80% accuracy on the ADHD dataset; achieved state-of-the-art results on the depression dataset AVEC 2014.
Lylath et al. 2022 [17]	Naïve Bayes, PCA, K-dimensional Clustering, ANN, SVM	Classification Accuracy, Cluster Evaluation	Early	Development of ASD detection methods; supports early intervention assessment.
S. Fazeli et al. 2023 [8]	Multi-modal self-supervised learning pipeline with modality-specific encoders (Bi-LSTM) and a modality importance head	Test accuracy (compared across training setups)	Late	Best generalisation performance achieved using late-fusion with supervised training and contrastive regularisation, yielding a test accuracy of 73%.
W. Lin et al. 2023 [18]	Multi-modal Deep Denoising Auto-Encoder + GMM-FV encoding + Random Forest (feature selection) + LR and MLP (final classification)	Recall	Hybrid	Successfully predicted depression/anxiety distress labels using multi-modal features; fidgeting behaviours contributed meaningfully to psychological distress prediction, with a recall of 75%.
Kennedy et al. 2024 [19]	Random Forest, Regression Analysis, Bayesian Harmonisation	*p*-values for group comparisons; association strength with verbal learning and memory (VLM) scores	Late	Strong VLM deficits found for dementia and schizophrenia (*p* < 0.001); no consistent association for depression and ADHD (*p* > 0.05); greater deficits with increased recall delay; sex and age differences observed in specific conditions.
Kautzky et al. 2020) [20]	Random Forest classifier with five-fold cross-validation and ten repeats	Mean accuracy, sensitivity, specificity	Early	Specific ROIs (posterior cingulate, cuneus, precuneus, central gyri) and SNPs (HTR2A rs1328684, rs6311; HTR1B rs130058) were highly discriminative; demonstrated feasibility of computer-aided diagnostic tools for ADHD; mean accuracy 0.82, sensitivity 0.75, specificity 0.86.
Andrikopoulos et al. 2024 [2]	Logistic Regression, k-Nearest Neighbors (KNN), Random Forest, Support Vector Machines (SVMs); best results from SVM	Accuracy, Sensitivity, Specificity	Early	SVM achieved 81.6% accuracy, 81.4% sensitivity, and 81.9% specificity; integration of all physiological signals yielded the best performance.
Mennon et al. 2020 [20]	Ensemble deep learning 3D CNN	Accuracy, Sensitivity, Specificity, F1 score	Early	Classification accuracy of 72%, sensitivity of 70%, specificity of 72%, and F1-score of 70%.
De Lacy et al. 2023 [22]	Deep learning architecture optimized using an AI algorithm for feature selection and classification	Accuracy, AUROC, Precision, Recall	Early	High accuracy in predicting ADHD, ODD, and CD onset (accuracy ∼86–97%, AUROC 0.919–0.996, precision/recall ∼82–97%); neural-only models showed reduced performance; parental aggressive traits predicted ODD; limbic MRI metrics predicted CD; sleep and school data were predictive across disorders.
McNorgan et al. 2020 [23]	Multilayer feedforward neural network (classifier models)	Accuracy, Cohen’s d, Sensitivity, Specificity	Hybrid	Functional connectivity during tasks predicted both ADHD diagnosis and behavioural task performance with 91% accuracy; key features involved visual, ventral attentional, and anterior default mode networks.
Muthuraman et al. 2019 [24]	SVM	Accuracy (98%), connectivity measures across frequency bands (alpha, beta, gamma)	Late	ADHD patients exhibited altered thalamo-cortical connectivity, primarily outflow from cortical regions, unlike controls with bidirectional connectivity; 98% classification accuracy achieved using combined features from five frequency bands.
Yoo et al. 2019 [25]	Random Forest classifier with five-fold cross-validation and ten repeats	Accuracy, AUC	Hybrid	Best accuracy of 85.1% using cortical thickness and volume features; key structural predictors included the insula, sensory/motor regions, and inferior frontal cortex.
Luo et al. 2020 [26]	Support Vector Machine (SVM) in a bagging method with 7 base classifiers	Area under the ROC Curve (AUC)	Late	Bagging-based ensemble learning with SVM achieved AUC of 0.89 for ADHD vs. controls and 0.90 for ADHD persisters vs. remitters; significant features included nodal efficiency in the right inferior frontal gyrus, right MFG–IPL functional connectivity, and right amygdala volume; higher nodal efficiency in MFG was linked to symptom remission.
Manjur et al. 2025) [27]	Random Forest (RF), Gradient Boosting (GB), Decision Tree (DT), SVM	Accuracy, Sensitivity, Specificity	Early	Combined feature model outperformed the time-domain-only model (65% accuracy), achieving 86% accuracy and 98% sensitivity using RF.
Yoo et al. 2020 [28]	Logistic Ridge Regression (LR), Support Vector Machine (SVM), J48, Decision Tree	LR: 95.5% accuracy (AUC 0.99); SVM: 91% (AUC 0.85); J48: 90% (AUC 0.87) on training data; J48: 86.1% accuracy (AUC 0.92) on validation data	Early	Key predictors included inattention symptoms (ADHD-RS), CPT response time variability, DAT1/ADRA2A/SLC6A2 polymorphisms, and fronto-striatal connectivity, with overall accuracy of 90–95%.
Weibe et al. 2024 [13]	Support Vector Machine (SVM)	Accuracy on independent test set	Early	EEG-based features did not contribute to prediction; the VR-based attention task was useful for ADHD differentiation, predicting ADHD with 81% accuracy.
Ma G et al. 2025 [29]	Logistic Regression for classification	AUC: 0.892, Sensitivity: 80.28%, Specificity: 84.51%	Early	ADHD patients showed lower frontal hemodynamic response during a verbal fluency task (fNIRS), higher errors and latency in an anti-saccade task, and more commission errors in the CPT; the multimodal approach yielded an accurate diagnostic tool.
Chaim-Avancini et al. 2017 [30]	Support Vector Machine (SVM)	AUC: 0.71 (ADHD vs. HC), 0.74 (males); Diagnostic Accuracy (DA): 66% (ADHD vs. HC), 74% (males)	Early	The SVM classifier yielded moderate discrimination between ADHD patients and controls, particularly among males.
Grazioli et al. 2021 [31]	Finite Gaussian Mixture Model (FGMM)	Bayesian Information Criterion (BIC), cluster assignment probability	Early	Identified two clusters across several clinical subscales (cognitive problems, ADHD index, perfectionism), reflecting heterogeneity in methylphenidate (MPH) response; neuropsychological reaction-time measures also showed two clusters.
Guigou et al. 2025 [32]	Multimodality Biometry System (AI pipeline), Linear Mixed-Effects Models	Restricted Maximum Likelihood (REML), R^2^, Log-Likelihood, Mean Absolute Error (MAE), Cohen’s d	Early	Captured multidimensional ADHD symptomatology; predicted working memory (*p* < 0.0001), processing speed (*p* < 0.0001), visuo-spatial skills (*p* = 0.003), inattention (*p* = 0.04), and achievement (*p* = 0.04); the multimodal model generally outperformed unimodal models for comprehensive assessment.
Firdous et al. 2023 [33]	Support Vector Machine (SVM) classifier with a non-linear kernel	AUC: 0.71–0.74, Diagnostic Accuracy (DA): 66–74%	Early	The neuroanatomical signature based on MRI and DTI data discriminated ADHD patients from healthy controls with AUC up to 0.74 and DA up to 74%; male-only subgroup analysis yielded stronger results.

## Data Availability

No new data were created or analyzed in this study. Data sharing is not applicable to this article.

## Supplementary Materials

The following supporting information can be downloaded at https://www.mdpi.com/article/10.3390/healthcare14193259/s1, Table S1: Qulity assesment of included studiesusing modified Newcastle Ottawa Scale.
