# Supplementary material for: Multimodal Machine Learning Approaches to Detect Attention-Deficit/Hyperactivity Disorder (ADHD) Using Physiological and Behavioural Biomarkers: A Systematic Review
Source: Healthcare (Basel). 2026 Oct 1;14(19):3259. doi: 10.3390/healthcare14193259 (PMC13665459; doi:10.3390/healthcare14193259)
Supplement: Supplementary file 1 [file healthcare-14-03259-s001.zip › healthcare-4531148-supplementary.pdf]

Supplementary Table S1. Quality assessment of included studies using a modified Newcastle-Ottawa Scale.

No	**Authors** **Year**	Q1	Q2	Q3	Q4	Q5	Q6	Q7	Q8	Q9	Total score (9%)
1	Nash et al.2024	Y	Y	Y	Y	Y	Y	Y	Y	Y	9
2	Zhang et al.2024	Y	Y	Y	Y	Y	Y	Y	NR	Y	8
3	Abdolmaleki.et.al.2020	Y	Y	NR	Y	Y	Y	Y	Y	Y	8
4	Shuanglin.et.al.2024	Y	Y	NR	Y	NR	Y	Y	Y	Y	7
5	Yichun.et.al.2024	Y	Y	NR	Y	NR	Y	Y	Y	Y	7
6	Lylath.et.al.2022	Y	Y	NR	Y	NR	Y	Y	Y	Y	7
7	S. Fazeli et al.2023	Y	Y	Y	Y	NR	Y	Y	Y	Y	8
8	W. Lin et al.2023	Y	NR	Y	Y	NR	Y	Y	Y	Y	7
9	Kennedy et al.2024	Y	Y	Y	Y	Y	Y	Y	Y	Y	9
10	Kautzky.et. el.2020	Y	Y	NR	Y	NR	Y	Y	Y	Y	7
11	Andrikopoulos.et.al.2024	Y	Y	Y	Y	Y	Y	Y	NR	Y	8
12	Kautzky et al.2020	NR	Y	Y	Y	Y	Y	Y	NR	Y	8
13	Lacy. et al.2023	NR	Y	Y	Y	Y	Y	Y	Y	Y	8
14	McNorgan et al.2020	NR	Y	Y	Y	Y	Y	Y	NR	Y	8
15	Muthuraman et al.2019	Y	Y	NR	Y	NR	Y	Y	Y	Y	7
16	Yoo.et.al.2019	Y	Y	NR	Y	NR	Y	Y	Y	Y	7
17	Lyo.et.al.2020	Y	Y	Y	Y	Y	Y	Y	NR	Y	8
18	Manjur.et.al.2025	Y	Y	NR	Y	NR	Y	Y	Y	Y	7
19	Yoo et al.2020	Y	Y	Y	Y	Y	Y	Y	Y	Y	9
20	Weibe et al.2024	Y	Y	NR	Y	NR	Y	Y	Y	Y	7
21	Ma G et al.2025	Y	Y	Y	Y	Y	Y	Y	Y	Y	9
22	Chaim-Avancini et al. 2017	Y	Y	Y	Y	Y	Y	NR	Y	Y	8
23	Grazioli et al.2021	Y	Y	NR	Y	NR	Y	Y	Y	Y	7
24	Guigou et al.2025	Y	Y	NR	Y	NR	Y	Y	Y	Y	7
25	Firdous et al.2023	Y	Y	Y	Y	Y	Y	NR	Y	Y	8

Key: Y= Yes; **NR**= Not reported, **NA**=Not appropriate

Question codes:

1. Was the sample frame appropriate to address the target population?

2. Were study participants sampled in an appropriate way?

3. Was the sample size adequate?

4. Were the study subjects and the setting described in detail?

5. Was the data analysis conducted with sufficient coverage of the identified sample?

6. Were valid methods used for the identification of the condition?

7. Was the condition measured in a standard, reliable way for all participants?

8. Was there appropriate statistical analysis?

9. Was the response rate adequate, and if not, was the low response rate managed appropriately?
